# Hypolipidemic Effects of β-Glucans, Mannans, and Fucoidans: Mechanism of Action and Their Prospects for Clinical Application

**DOI:** 10.3390/molecules25081819

**Published:** 2020-04-16

**Authors:** Tatiana A. Korolenko, Nataliya P. Bgatova, Marina V. Ovsyukova, Alexandra Shintyapina, Vaclav Vetvicka

**Affiliations:** 1 Department of Clinical Neuroscience, Behavior and Neurotechnologies, Institute of Physiology and Basic Medicine, Timakov St. 4, Novosibirsk 630117, Russia; t.a.korolenko@physiol.ru (T.A.K.); maryov@ngs.ru (M.V.O.); 2 Laboratory of Ultrastructural Research, Department of Experimental Pharmacology, Research Institute of Clinical and Experimental Lymphology—Branch of the Institute of Cytology and Genetics, Siberian Branch of Russian Academy of Sciences, Novosibirsk 630117, Russia; nataliya.bgatova@yandex.ru; 3 Institute of Molecular Biology and Biophysics, Federal Research Center, Timakov St. 2, Novosibirsk 630117, Russia; shintyapina@yandex.ru; 4 Department of Pathology, University of Louisville, Louisville, KY 40292, USA

**Keywords:** polysaccharide, hypolipidemic effect, β-glucan, fucoidan, mannan

## Abstract

The search for lipid-lowering drugs is important for clinical medicine. This review summarizes our research findings regarding the hypolipidemic activity of polysaccharides. There are several validated agents altering lipid levels which reduce the risk of atherosclerotic cardiovascular events. Nonetheless, for many people, the risk of such an event remains unacceptably high despite treatment with these agents. This situation has prompted the search for new therapies to reduce the residual cardiovascular risk. The lipid-lowering effect of β-glucans consumed with food was demonstrated in patients with atherosclerosis. The mechanism of the protective effect of β-glucans is poorly studied. The effects of β-glucans are mediated by Toll-like receptors, by dectin-1, and possibly by other receptors. Nevertheless, the mechanism of the protective action of β-glucan in lipemic mice has been studied insufficiently. This review will present up-to-date information regarding experimental hypolipidemic polysaccharide compounds that hold promise for medicine. Phagocyte-specific chitotriosidase in humans contributes to innate immune responses against chitin-containing fungi. This enzyme has been first described in patients with Gaucher disease and serves as an important diagnostic biomarker. It has been reported that, in mice, chitin particles of certain size are recognized by macrophages through Toll-like receptors, dectin-1, and to a lesser extent through mannose receptor.

## 1. Introduction

The search for lipid-lowering drugs and biologics is important for the development of pharmacology and for clinical medicine, as coronary heart diseases remain a major health problem in developed countries. Barter and Rye have stated that, among the drugs currently under development, there are two categories: (1) those that target atherogenic lipoproteins and (2) those that target potentially cardioprotective high-density lipoproteins [1]. Statin therapy is important for the treatment of atherosclerosis, dyslipidemias, type 2 diabetes, and many cardiovascular diseases. Nonetheless, adverse effects of statin therapy are being increasingly recognized; among them, muscle-related symptoms are rather common [2,3]. Treatment with statins can result in liver function changes secondary to the development of steatohepatitis, especially during the treatment of dyslipidemia in patients with type 2 diabetes [4]. The search for new hypolipidemic compounds is currently relevant in pharmacology and clinical medicine, particularly for some able to overcome limits of current lipid-lowering pharmaceuticals. In this regard, the topic of polysaccharides with hypolipidemic effects is of interest. Herein, we present some experimental data on the hypolipidemic activity of several polysaccharides that have potential use in clinical practice. According to recent review [5], natural polysaccharides reduce triglyceride level through ATGL-(PPAR-α)/(PGC-1α), 24 (SREBP-1c)-ACC/FAS and ACC-CPT1 signal pathways, and exert cholesterol lowering effects via (SREBP-2)-HMGCR and bile acid biosynthesis pathways. Activation of adenosine monophosphate-activated protein kinase (AMPK) is the key factor that mediated the simultaneous regulation of both glucose and lipid metabolisms by polysaccharides.

## 2. Experimental Models of Hyperlipidemia Used to Study Hypolipidemic Effects of Polysaccharides

In general, we used two models of experimental dyslipidemia (with different doses of the key agents) in our research: dyslipidemia induced by nonionic detergent Triton WR 1339 and dyslipidemia induced by poloxamer 407 (P-407). The latter model was developed by Johnson, and its properties are summarized in a review of the 25-year use of this dyslipidemia model [5]. Triton WR 1339 is a nonionic detergent, isooctylpolyoxyethylene phenol, with the common formula (C_14_H_22_O⋅C_2_H_4_O⋅CH_2_O)_n_, whereas P-407 (Pluronic F-127) is a nonionic block polyoxyethylene polyoxypropylene copolymer (molecular weight 9840–14,600), with the general formula HO(C_2_H_4_O)_a_(C_3_H_6_O)_b_(C_2_H_4_O)_a_H, where a = 100 and b = 65. The two dyslipidemia models are highly similar, but changes in serum atherogenic fractions are more pronounced under the influence of P-407 (Table 1). A specific feature of the model based on P-407 (compared to Triton WR 1339) is elevated concentrations of anti-atherogenic high-density lipoprotein (HDL)-C and HDL-TG (subfractions HDL2 cholesterol and HDL2 triglycerides).

Each of these dyslipidemia models has advantages and disadvantages in terms of their ability to reproduce the human pathology. The positive side of these models is their reproducibility, dose-dependent hyperlipidemia in small laboratory animals (rats and mice), possibility of several models of the pathology (to study subtypes of dyslipidemia via single or repeated modes of administration of the agent), and feasibility of an atherosclerosis model in mice as a result of repeated administration of P-407 for 4–6 months. Dyslipidemia models created by means of Triton WR 1339 or P-407 in mice do not involve significant toxicity of these agents in vivo (i.e., adverse effects). Moreover, the mechanism of hyperlipidemia has been actively studied, and this knowledge is important for the development of experimental therapies for dyslipidemia. It is noteworthy that, with both agents, substantial hypertriglyceridemia is reproduced as well [6]; this finding is important for the research on the pathogenesis of atherosclerosis [7].

## 3. Dyslipidemia Models

**Figure 1:**
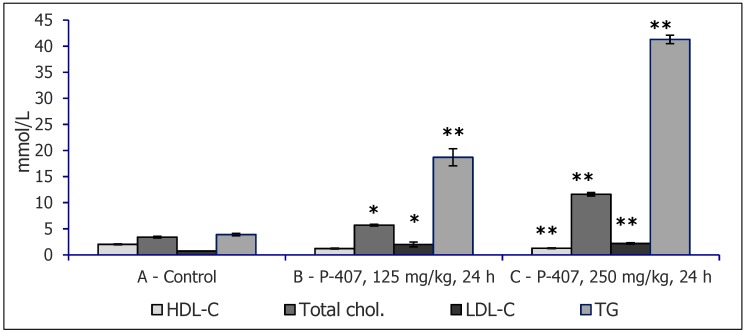
The serum lipid profile of untreated CBA mice and CBA mice with acute lipemia induced by different doses of poloxamer 407, mmol/L (mean ±SD). * *p* < 0.01 vs. control; ** *p* < 0.001 vs. control.

**Figure 2:**
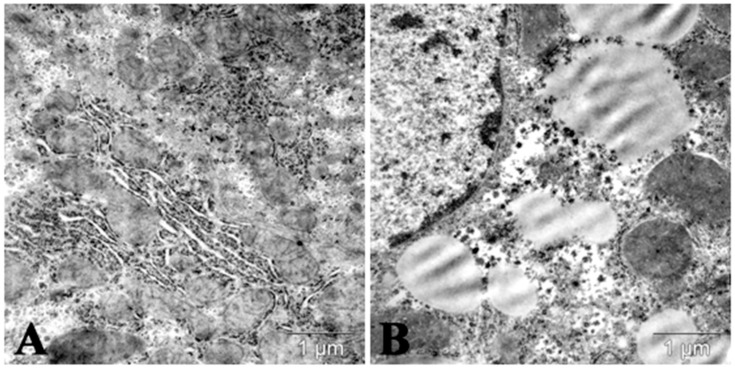
Lipid droplets in cytoplasm of mice hepatocyte in acute lipemia induced by the repeated administration of P-407 (300 mg/kg, twice per week during 30 days. (**A**) Control; (**B**) 24 h after the last administration of P-407.

With respect to these agents, dose dependence of hyperlipidemia was shown in CBA and ICR male and female mice receiving different doses of P-407: 250, 300, or 500 mg/kg, intraperitoneally (i.p.), in a single injection (Figure 1). The advantage of the P-407 model is the absence of cell membrane damage; P-407 causes no erythrocyte lysis and can be injected repeatedly for atherosclerosis simulation [5]. Changes in serum lipids in the dyslipidemia induced by injections of Triton WR 1339 or P-407 are similar to those observed in types 2a/2b and 3 of human dyslipoproteinemia. The mechanisms of dyslipidemia development in response to injections of Triton WR 1339 or P-407 into mice have also been studied. It has been found that this dyslipidemia is related to serum lipoprotein lipase inhibition and, hence, to disorders of very low-density lipoprotein (VLDL) clearance. Serum concentrations of total cholesterol and TG increase 24 h after injections of P-407 or WR 1339, reaching rather high levels, higher than the respective parameters in patients with atherosclerosis. In general, either agent, P-407 or Triton WR 1339, can be used for lipemia modeling and testing of hypolipidemic drugs, including triglyceridemia-reducing agents. Increased number of lipid droplets were noticed in liver of mice receiving P-407 (Figure 2).

In terms of dyslipidemia, there are similarities in serum lipid level changes seen in the mice receiving either Triton WR 1339 or P-407 in the same dose and mode of administration (500 mg/kg, i.p. injections, assessment 24 h later) [6]. This experimental hyperlipidemia includes a drastically increased level of total cholesterol and especially TG when compared with untreated mice (receiving saline; Table 1). Normalization of the levels of lipids under study occurred up to the 10th day after the model initiation. In a study on the P-407 model, evaluation of the lipoprotein fraction and subfraction composition by small-angle X-ray scattering on a diffractometer revealed elevation of the serum concentrations of anti-atherogenic HDL cholesterol (HDL-C) and HDL-TG (subfractions HDL2-CH and HDL2-TG) [6].

To study early atherosclerosis, a model based on P-407 intraperitoneal injections and involving a dose of 300 mg/kg twice a week during 1 month was developed. The mice were killed 24 h and 4 and 10 days after the last P-407 injection. At 24 h after the last P-407 injection in CBA mice, there was an increase in systolic and diastolic blood pressure as well as hyperlipidemia with increased levels of serum total cholesterol, TG, and atherogenic LDL-C [8]. At 24 h after the last administration of P-407 in mice, there was a drastic increase in total serum cholesterol, atherogenic non-HDL cholesterol, and especially the total TG level. Behavioral changes included increased anxiety according to the elevated plus maze test. Later, on the 5th day after the last P-407 administration, elevated levels of total cholesterol and non-HDL cholesterol were still present (in contrast to the group with a single dose of P-407, where normalization of these indices was observed), and serum TG concentration decreased sharply but did not reach the normal values. Morphological changes observed in the P-407–treated mice included contractile-type changes in cardiomyocytes; in the liver, numerous foamy macrophages, zones of disappearance of cellular organelles as well as cholestasis were noted. In the heart, P-407 significantly increased the activity of cysteine (at 24 h) and of aspartate protease cathepsin D (at 24 h and 5 days after the administration). Thus, repeated administration of P-407 to mice induced increased anxiety typical for early atherosclerosis development (secondary to sustained dyslipidemia and the emergence of foamy macrophages in the liver) as well as modulated the activity of heart and liver aspartic protease cathepsin D.

In a model of atherosclerosis development via repeated administration of P-407 to mice for 4 months, we noted increased diastolic blood pressure and drastic upregulation of total serum cholesterol and especially TG [9]. P-407 significantly increased the levels of atherogenic LDL-C, intermediate-density lipoprotein (IDL) cholesterol, and VLDL-C. Additionally, we demonstrated increased serum CHIT1 activity (twofold higher compared to the control). In the heart of these mice, we observed increased cathepsin B and cathepsin D activities, and in the liver, higher cathepsin B activity. Electron-microscopic analysis revealed dystrophic changes in cardiomyocytes, and in the liver, there were dystrophic changes of hepatocytes with an increased number of autophagosomes and lysis of some cells. The repeated administration of P-407 to mice induced atherosclerosis in the heart and lipid storage syndrome in liver macrophages secondary to sustained dyslipidemia and the formation of foamy macrophages in the liver.

## 4. β-d-Glucans

β-d-Glucans (β-glucan henceforth), generally called biological response modifiers, belong to a heterogeneous group of natural physiologically active compounds found in plants, seaweed, fungi, and microbes [10]. Various physicochemical characteristics, including molecular weight, primary structure, solubility, and branching, affect their biological activities but no final correlation between the structure and function has been found [11,12]. In general, their primary structure mostly depends on their origin and type of isolation. Originally, interest focused on the role of β-glucans in various immune reactions. After several decades of intensive research, β-glucans effects have been well established, and β-glucans have been found to influence immune reactions in every species tested, from earthworms to humans [13]. β-Glucans have been found to positively influence cellular immunity (phagocytosis, NK cell activity, wound healing) as well as humoral immunity (antibody response, cytokine production), resulting in well-documented stimulation of anti-infective immunity, anti-parasite immunity, and anti-cancer immunity [14,15,16,17].

The mechanisms of action start with recognition by specific receptors such as CR3, dectin-1, lactosylceramides, and TLRs [18,19]. Full details of the molecular mechanisms are still not fully established, but NF-κB activation, phosphorylation of JNKm, p38 MAPKs and ERK, Syk phosphorylation, PI2K pathway, and ERK1/2 pathways have been reported [20,21,22,23,24].

In addition to their well-established positive effects on modulation of various aspects of immune reactions, β-glucans have attracted attention as potential nutraceuticals to decrease cholesterol. Most of the original studies were performed on mouse and rat models. However, lipid-lowering effects of β-glucan were also confirmed in broiler chicks and hamsters [25,26]. Based on these successful findings, numerous human trials followed. This interest has been potentiated by the fact that the US agency FDA has endorsed the connection between the suppression of serum cholesterol and increased dietary fiber uptake for both psyllium and oat fiber [27,28]. Originally, the running hypothesis suggested that only soluble glucans working as soluble fibers were able to lower levels of cholesterol and lipids and that the insoluble fibers only participated in fecal bulking [29]. The proposed mechanisms of action were binding to free fatty acids, bile acids, and cholesterol. Later, this hypothesis was rejected and the research revealed that the effects of β-glucan were dependent on fermentation capability and on the viscosity [30]. Some studies suggested entrapping the bile acid micelles increasing elimination of cholesterol and fat, leading to lower levels of cholesterol in the liver and subsequent compensation from serum [31].

Meta-analysis of the effects of β-glucan supplementation usually revealed solid decrease of total and HDL-cholesterol, but offer no real insight to the source, purity, or period of supplementation, making these studies less relevant and the search for underlying mechanisms more difficult [32].

Most studies focused on barley-derived β-glucan. This was mostly based on the study showing that barley cultivars rich in fiber have significant hypocholesterolemic effects in men [33]. As results of some of the human studies were not fully consistent, most probably due to the different purities and doses of β-glucan used, a meta-study analyzing 8 clinical trials found significant lowering of triglycerides, LDL-cholesterol, and total cholesterol levels, but no changes in HDL-cholesterol levels [34]. A study using mildly hypercholesteromic people supplemented with oat β-glucan showed 10% decrease of total cholesterol and 15% decrease of LDL-cholesterol [35]. Similar effects are summarized in the review by Hermansen et al. [36]. Conversely, a randomized study showed variable results in the model of mildly hypercholesteromic men, but this lack of effects can be ascribed to the use of glucan-enriched barley instead of purified β-glucan [37]. Recent systematic meta-analysis of 14 clinical trials showed that barley β-glucan decreased LDL cholesterol [38].

A study focused on the possible effect on fecal bile acid excretion in human patients after 5-week supplementation found increased fecal SCFA concentrations and bile acid excretion, suggesting modifications of intestinal microbiota as a response to β-glucan treatment are responsible for cholesterol-lowering effects of β-glucan [39]. This study was a follow-up of the previous investigation which found that β-glucan supplementation caused interruption of bile acid metabolism [40]. Both studies strongly suggested that the original idea of inhibiting cholesterol synthesis and/or absorption is probably wrong. This hypothesis for further supported by findings of short chain fatty acids composition and alteration in gut microbiota with decrease of diversity in the glucan-supplemented group [41]. Other effects were found in rats fed with high levels of cholesterol. Barley-derived β-glucan of various molecular weights produced different effects on microbial composition and cecal fermentation, but had no effects on blood cholesterol levels [42]. Some studies suggested that the lipid-lowering effect is promoted by excretion of fecal lips and by regulation of the activity of hydroxyl-3-methyl-glutartyl-coenzyme A reductase [43].

A detailed study of β-glucan and oat structure in lipid digestion in vitro demonstrated that it is a complex process which might be more dependent on oat structure and physicochemical properties than on β-glucan alone, which might explain the differences among various studies using oat products and oat-derived β-glucans [44]. In addition, studies showing only mediocre effects might not only show the possible relation of these effects to some particular apolipoprotein E phenotype but, due to very low level of β-glucan (only 16.6%), the conclusions are difficult to reach [45]. A study using dogs found significant reduction of total cholesterol, LDL- and VLDL-cholesterol concentrations but the effects required rather high concentration of 10 g/kg of food, making these effects nonphysiological [46].

An interesting study focused not only on the possible effects of oat-derived β-glucan in people with risk of metabolic syndrome, but also evaluated the importance of composition of glucan-enriched food with three different food matrixes: dairy, eggs, and bakery. The data showed promising effects of glucan on lowering HDL-cholesterol and TG levels, but these effects differed based on the used food [47].

Using a yeast-derived β-glucan and ob/ob mice model, Cao et al. found that oral supplementation promoted glucose and lipid homeostasis in the liver, probably via downregulation of genes responsible for gluconeogenesis, cholesterol synthesis, and biosynthesis of fatty acids [48]. This study not only showed significant effects of yeast β-glucan, but also showed that, as long as the purity and biological activity of β-glucan was significantly high, there was no difference between the sources of β-glucan. Our own study compered four different glucans from several different sources (yeast and grain) and found that they all induced similar effects, but the strength of these effects differed widely from one glucan to another [49]. Four weeks of supplementation with yeast-derived β-glucan fiber significantly lowered total cholesterol level in hypercholesterolemic men [50].

These results were confirmed on a human model of patients with diabetic retinopathy, where a 3-month β-glucan supplementation also resulted in significant reduction of total cholesterol levels and improved levels of HDL-cholesterol [51]. This model is particularly interesting due to its close relationship with obesity, adipokines, and leptin.

Very little research has been done on mushroom-derived β-glucans. One study suggested that dried mushroom powder containing an unknown amount of β-glucan changes lipid metabolism by inhibiting the accumulation of liver lipids [52]. However, the mechanisms are unknown and, as it is not established if any action of mushroom powder can really be attributed to β-glucan, these results remain questionable.

Similarly less studied are the effects of β-glucans isolated from *Euglena gracilis*. Using a model of diet-induced obese mice, food supplementation for 74 days resulted in improvements in LDL-cholesterol levels with suggested induction of β-oxidation and changes in fatty acid metabolism [53]. The high doses (5%) necessary to see these effects makes the conclusions nonphysiological, despite promising effects against obesity.

The precise mechanisms of β-glucan action in lowering cholesterol and other lipids are still not fully established. The result variances could be secondary to differences among individual glucans and their purity. Clearly, more comparative studies are needed. For a current review discussing all aspects of β-glucan-cholesterol relations, see Sima, Vannucci and Vetvicka [29].

### Hypolipidemic Effects of Carboxymethylated β-Glucan (CMG) and Statin (Atorvastatin) in Acute Lipemia in Mice

First, we showed a hypolipidemic effect of atorvastatin at a relatively high dose (75 mg/kg) in the murine model of hyperlipemia induced by Triton WR 1339 [54]. In Triton WR 1339–treated mice (500 mg/kg, 24 h), there was a drastic increase in the atherogenic LDL-C fraction, an IDL-C subfraction, and VLDL-C fractions (VLDL-C3-5 subfraction). Treatment of the lipemia with atorvastatin resulted in the normalization of the atherogenic LDL-C fraction and of the IDL-C subfraction. A decrease in VLDL-C (VLDL3-5-C subfraction) in total cholesterol and especially TG concentrations was demonstrated, too. Similar results were obtained with TG lipoprotein fractions and subfractions. According to our results and data from literature, the pleiotropic effects of atorvastatin in mice includes hypolipidemic anti-inflammatory effects (CRP), autophagy stimulation, and influence on cysteine proteases and matrix metalloproteases.

**Figure 3:**
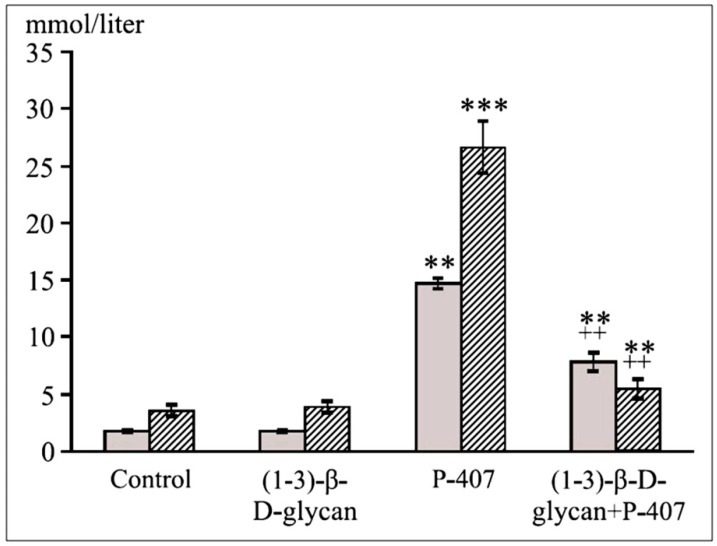
Hypolipidemic effects of (1–3)-β-d-glucan. A significant (*p* < 0.01) decrease in serum cholesterol and TG concentrations in the mice with acute lipemia. ** *p* < 0.01 vs. control; *** *p* < 0.001 vs. control; ++ *p* < 0.01 vs. the P-407 group; 7–10 mice per group.

We then compared the hypolipidemic effects of atorvastatin and of CMG (Institute of Chemistry, Slovak Academy of Sciences, Bratislava, Slovakia) in the model of dyslipidemia induced by P-407 (500 mg/kg, ip) in ICR and CBA mice [55]. Pretreatment with atorvastatin (75 mg/kg, 3 and 24 h before P-407 administration) had a significant hypolipidemic effect on the model of dyslipidemia induced by P-407 in ICR mice [56]. The statin at the above dose decreased both total cholesterol and TG levels but not to the control levels. Pretreatment with CMG (twice at a dose of 25 mg/kg, before P-407 administration) also exerted hypolipidemic action (a decrease in the TG level, *p* < 0.01, with a decrease tendency of total cholesterol). Hypolipidemic effect was shown in mice with acute lipemia pretreated by β-glucan (Figure 3). In general, the hypolipidemic influence of β-glucan or CMG was less potent than that of atorvastatin. Some biological activities (such as antioxidation) of β-glucan can be enhanced through carboxymethylation [57].

CMG primarily decreased the serum TG level and was not as effective as atorvastatin [56]. P-407 increased the level of atherogenic IDL-C [and LDL (1–3)-C subfractions] and VLDL-C fractions [VLDL(1-2)-C and VLDL(3–5)-C subfractions], with an increase in the total anti-atherogenic HDL-C fraction [HDL(2)-C subfraction]. In general, high-dose atorvastatin used in the study as pretreatment exerted its lipid-lowering and pleiotropic effects at the early stages of the acute lipemia induced by P-407 in the mice [56]. These results are in agreement with an older study showing that glucans not only inhibit intestinal uptake of long-chain fatty acids, but also downregulate some genes important for lipogenesis [58].

**Figure 4:**
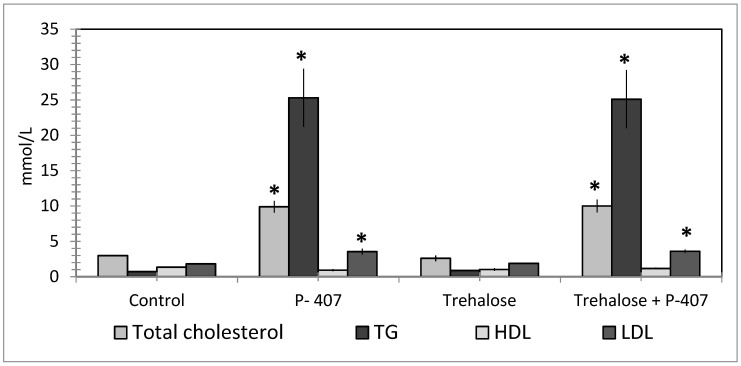
The serum lipid profile of mice with acute lipemia that were drinking a 2% solution of disaccharide trehalose for 10 days before P-407 administration. * *p* < 0.01 as compared to the control.

**Figure 5:**
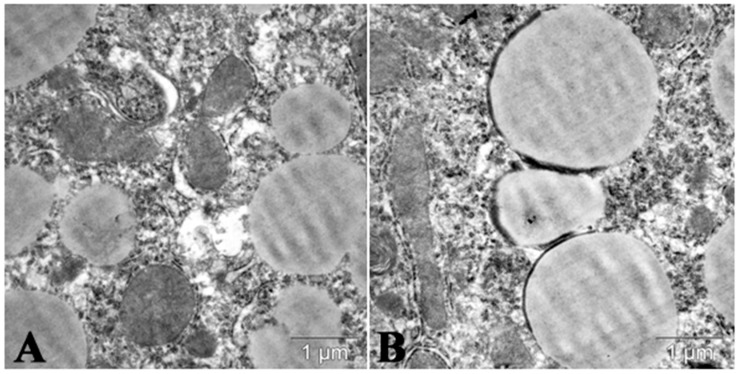
Lipophagy in cytoplasm of hepatocyte of mice with P-407-induced lipemia pretreated by trehalose (drinking 2% trehalose during 10 days). (**A**) Lipid droplets in liver cells in lipemia induced by P-407 (300 mg/kg, ip, single). (**B**) Forming of autophagosomes near and around lipid droplets in liver of mice with P-407-induced lipemia pretreated by trehalose (drinking 2% trehalose during 10 days).

Disaccharide trehalose had no effect on serum lipids during the acute lipemia induced by P-407 (300 mg/kg) in mice (Figure 4). Nonetheless, trehalose manifested a hypoglycemic effect in this experiment by significantly (*p* < 0.05) decreasing the level of blood glucose. Trehalose pretreatment was followed by increased autophagy in liver of mice with lipemia (Figure 5).

## 5. Mannans

Mannan (a polymer of mannose) is a constituent of the cell wall of higher plants, as is the hemicellulose type, glucomannan. Hemicellulose includes linear or branched polymers of sugars such as d-mannose, d-galactose, and d-glucose [59]. Evaluation of antioxidant and antimutagenic effects of yeast cell wall mannans (extracellular and cellular glucomannan from *Candida utilis*, mannan from *Saccharomyces cerevisiae*, and mannan from *Candida albicans*) revealed an antimutagenic effect mediated by different mechanisms of action; therefore, they hold promise as natural protective antimutagenic agents [60].

Notably, mannan was found to have antiviral activity related to mannan-binding lectin (MBL) [61]. In mice, MBL deficiency does not affect antibody production [62]. MBL is a key receptor in the innate immune system and is present in blood plasma; MBL is produced in the liver in response to infection and belongs to the group of acute-phase proteins [63,64]. MLB binds to specific carbohydrate motifs on the membranes of various pathogens, followed by stimulation of the immune system via the lectin pathway [65]. It has been shown that many pathological processes of common gastroenterological diseases (infection, immunological damage, and carcinogenesis) are linked to MBL [66]. Therapeutic use of MBL for MBL-deficient patients with a respiratory tract infection is potentially possible [67]. *MBL2* gene polymorphism is associated with various inflammatory diseases (without consistent changes in MBL serum levels); therefore, further elucidation of the role of MBL in these diseases is needed [63].

### Hypolipidemic Effects of Mannans

Recently, various polysaccharides isolated from algae, mushrooms, yeast, and higher plants caught the attention of researchers in the field of nutrition and medicine [68]. The reasons include their low toxicity, rare and mild adverse effects, relatively low cost, and a broad spectrum of therapeutic actions. The two best-studied polysaccharides are β-glucan and mannan [69]. Polysaccharides were shown to stimulate macrophages [70,71,72].

**Figure 6:**
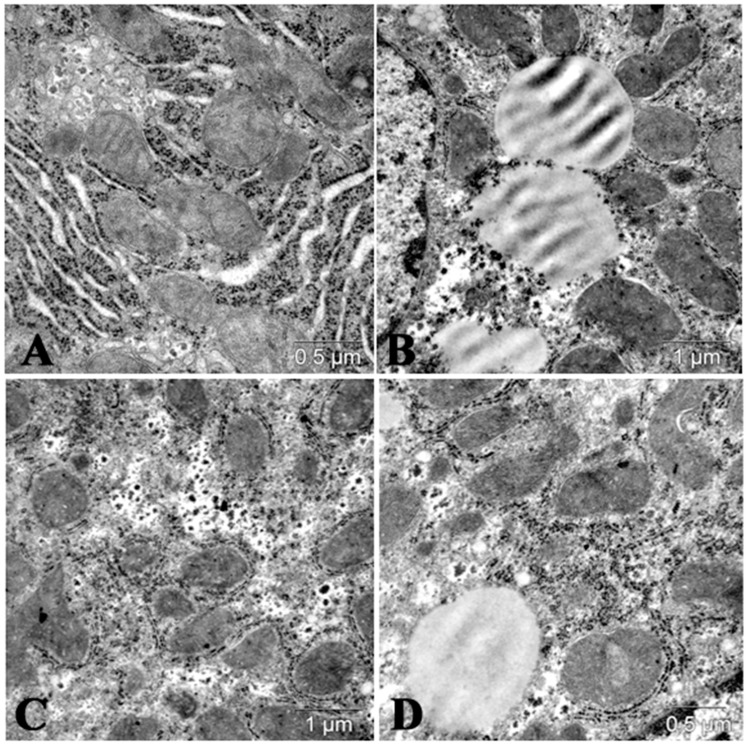
Hypolipidemic effects of mannan Serotype A and mannan Serotype B (*C. albicans*) in mice with lipemia induced by P-407. (**A**) Liver of control mice; (**B**) 24 h after P-407 administration in mice. Lipid droplets in cytoplasm of hepatocytes; (**C**) significantly decreased number of lipid droplets in liver cells of mice with lipemia induced by P-407 (24 h), pretreated by mannan A; and (**D**) moderate number of lipid droplets in liver of mice with P-407-induced lipidemia pretreated by mannan B.

Mannans, which are biological macromolecules of the polysaccharide class, function as an immunomodulatory and have been reported to stimulate macrophages in vivo via an interaction with mannose receptor [73]. Thus, they can be used to stimulate macrophages in order to effectively remove circulating atherogenic lipoproteins. In a recent study, our primary aim was to evaluate the hypolipidemic potential of mannans from *C. albicans* serotype A (mannan A) and serotype B (mannan B) in a murine model of hyperlipidemia [8,73]. Mannan A and mannan B were found to significantly stimulate (*p* < 0.05) both the proliferation of (*p* < 0.05; and nitric oxide production in) murine peritoneal macrophages in vitro. Pretreatment of CBA/Lac mice with mannan A prior to induction of hyperlipidemia significantly (*p* < 0.001) reduced serum atherogenic LDL-C, total cholesterol, and TG. Mannan B exerted a similar, but more potent, hypolipidemic action. Electron-microscopic analysis of the liver uncovered a significant (*p* < 0.001) decrease in the volume of lipid droplets when the hyperlipidemic mice were pretreated by either mannans (Figure 6). It was shown that in early atherosclerosis, mannan-binding lectin and C1q can play a protective role, reducing levels of free cholesterol accumulation in monocytes and monocyte-derived macrophages containing oxidized LDL, and thus presenting a new mechanism for removal of atherogenic LDL.

In conclusion, our findings suggest that both polysaccharide-based biological macromolecules evaluated in that study, namely, natural immunomodulators mannan A and mannan B, may function as effective lipid-lowering agents and could serve as an adjunct therapy with conventional hypolipidemic medication such as a statin.

Torrecillas et al. determined the effect of two doses of dietary supplementation with mannan oligosaccharides derived from the outer cell wall of a special strain of *S. cerevisiae* (Bio-Mos) on the growth, feed utilization, immune status, and disease resistance of European sea bass (*Dicentrarchus labrax*) [74]. The authors found statistically significant changes in the phagocytic index after supplementation with Bio-Mos. A positive correlation was found between the levels of lysozyme and alternative complement pathway activities in the blood and the dose of supplementation. In general, the results showed that dietary supplementation with MOS at 0.4% enhances sea bass growth, activates its immune system, and increases its resistance to a bacterial infection caused by direct inoculation into the gut, one of the main sites of infection in fish.

## 6. Fucoidans

Fucoidans are water-soluble complex polysaccharide mainly containing l-fucose and sulfate groups found mostly in brown seaweed and sea cucumbers. This polysaccharide is believed to have several promising therapeutic properties [75,76]. It can be found mostly in marine brown algae and echinoderms [77,78]. Possible therapeutic actions of fucoidans include antioxidant, antitumor, antimetastatic anticoagulant, antithrombotic, immunoregulatory, antiviral, and anti-inflammatory activities [79,80]. In addition, fucoidans were found to influence vascular physiology, inflammation, and oxidative stress [81]. Some new biological effects of fucoidan and the relation between its structure and anticancer activity were demonstrated recently, particularly when used as a part of nanomedicine [82,83]. Fucoidans are considered to be a promising therapeutic agent and the research has increased dramatically, resulting in discussion the potential use of fucoidans as therapeutic agents for atherosclerosis [84,85]. Suggested mechanisms of action include stimulation of secretion of various cytokines via activation of the PI3K/AKT pathway [86].

A direct comparison of six different fucoidans found strong potentiating effects on various immune reactions including phagocytosis, antibody formation, and NK cell activity, all of which participated in suppression of cancer growth [87]. However, these activities fluctuated among individual samples. At present, possible new benefits of fucoidan for the development of pharmaceutical dosage forms are being discussed in the literature [88]. For a recent review of biologic and therapeutic effects of fucoidans focused on anti-tumor, anti-inflammatory and anti-oxidant effects, see Luthuli et al. [80].

Compared to β-glucan and mannan, the effects of fucoidans on lipids are much less studied, possibly due to the significant differences between fucoidans isolated from different sources, caused by often unknown amount of additional molecules such as d-xylose, l-rhamnose or acetyl groups [89]. However, some data suggested possible effects as an anti-obesity treatment via anti-adipogenic activity, downregulating key markers such as adipocyte protein 2, and via inhibition of lipid accumulation [90].

Various animal experiments suggested that fucoidan treatment results in reduction of total cholesterol, LDL-cholesterol, and triglyceride levels with an increase of the HDL-cholesterol levels and activity of hepatic lipoprotein [91,92].

Compared to glucan and mannan, fucoidan studies were often more focused on possible mechanisms of action. In vitro experiments found suppression of genes responsible for acetyl CoA carboxylase, fatty acid binding proteins, and peroxisome PPARγ [93]. Fucoidan supplementation resulted in reduction of weight gains and fatty liver deposits, suggesting changes/increase in lipid metabolism and confirming the previous in vitro results [94]. Another study found that fucoidan supplementation improved levels of serum lipids via hepatic SREBP-2-mediated action [95]. Another possible mechanism includes modulation of Wnt/β-catenin pathway [96].

A different approach, using mice subjected to high fat diet, showed that 8-week supplementation lowered not only both serum cholesterol and LDL-cholesterol, but also liver cholesterol levels. In addition, fucoidan improved liver steatosis, lowered expression of cholesterol-related proteins, and changed gut microbiota [97].

Some findings demonstrated strong hypolipidemic effects which were comparable to those of lovastatin with the possible mechanism being improved activity of LCAT [98]. An interesting recent study found that fucoidan can inhibit synthesis of cholesterol by downregulation of HMG-CoA-R and by upregulation of LCAT. In addition, downregulation of SREBP-1c resulted in lower synthesis of fatty acids, which was also found in an independent study using a different type of fucoidan [85,99].

**Figure 7:**
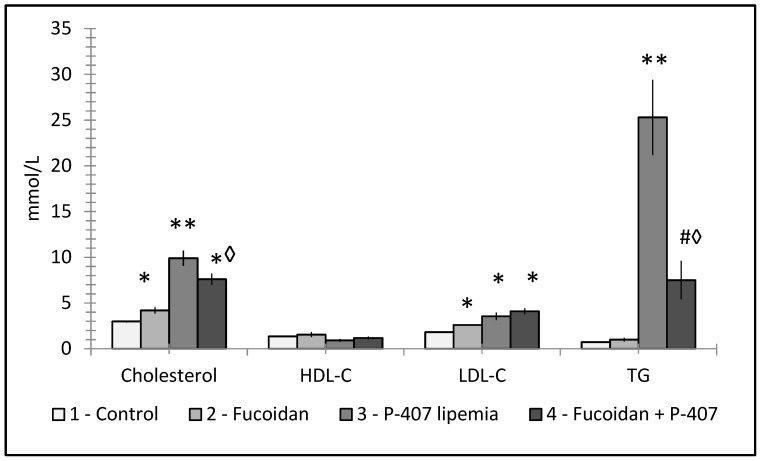
Effect of fucoidan pretreatment on serum lipids of C57Bl/6 female mice with acute lipemia induced by poloxamer 407 (250 mg/kg, ip, 24 h after). * *p* < 0.05; *◊ *p* < 0.01; ** *p* < 0.001 vs. control. #◊ *p* < 0.001 vs. P-407.

A detailed study evaluated the effects of fucoidans used in two different routes (parenteral and peroral) on lipid metabolism in mice with P-407-diduced dyslipidemia. Fucoidan treatment ameliorated all increases in VLD-cholesterol, total cholesterol, and TG levels caused by P-407. In addition, strong anti-inflammatory effects were found [76]. In both cases, no differences in the effects based on the delivery route were found. Figure 7 shows the prophylactic effects fucoidan supplementation had on serum lipids in experimentally-induced lipemia.

Very few human subject studies focused on fucoidan effects. However, even using a human model of obese patients, clinical trials found lower LDL-cholesterol levels after 3-month supplementation with fucoidan [100]. A recent observation focused on reverse cholesterol transport as a possible target of fucoidan action. Using mice exposed to high-fat diet model, fucoidan supplementation strongly reduced total cholesterol and TG levels. In the liver, fucoidan increased expression of several important receptors such as liver X receptor or PPARα and PPARγ. In the small intestine, fucoidan increased cholesterol excretion and decreased cholesterol absorption. Altogether, fucoidan treatment modulated protein related to reverse cholesterol transport [101]. It seems that fucoidans affect lipids by different mechanisms than β-glucans and mannans. However, due to numerous significantly different types of fucoidans, the full elucidation of mechanisms of action is still far from clear.

## 7. Autophagy/Lipophagy Induction and Lipid Metabolism Changes Induced by Polysaccharides

Recently, outstanding autophagy studies have been registered. In general, autophagy presents the lysosomal degradation of cytosolic material in cells, but the investigation of lipophagy has yet to fully develop [102]. Lipophagy, the autophagic degradation of intracellular lipid droplets (LD), plays an important role in lipid metabolism in vivo [103]. It was reported that hypolipidemic effects of some natural polysaccharides in hepatocytes during liver steatosis treatment involved autophagy activation/lipophagy [104,105]. Mechanisms of hypolipidemic effects of natural polysaccharides via induction autophagy were analyzed in the comprehensive review by Wu, et al. [106].

Investigation of lipophagy regulation will open new targets for therapeutic approaches, which are useful against lipid metabolism disorders in obesity, liver disease, and other diseases. In addition, lysosomal acid lipase (LAL), the enzyme that facilitates lipophagy, has been found to exhibit tumor suppressor activity.

The physiological importance of lipophagy has been demonstrated in mammalian cell types, revealing that this pathway has additional functions to supplying free fatty acids to maintain cellular energy stores [107]. Recent studies have provided new insights into the underlying mechanisms of lipophagy induction as well as the consequences of lipophagy on cell metabolism and signaling.

The recent findings that LD can be selectively degraded by the lysosomal pathway of autophagy through a process termed lipophagy has opened up a new understanding of how lipid metabolism regulates cellular physiology and pathophysiology [108]. Many new functions for autophagic lipid metabolism been defined in various diseases including liver disease. Lipophagy was originally described in hepatocytes, where it is critical for maintaining cellular energy homeostasis in obesity and metabolic syndrome. In vitro and in vivo studies have demonstrated the selective uptake of LDs by autophagosomes, and inhibition of autophagy can reduce the β-oxidation of free fatty acids due to the increased accumulation of lipids and LD. The identification of lipophagy as a new process dedicated to cellular lipid removal has mapped autophagy as an emerging player in cellular lipid metabolism. Pharmacological and/or genetic modulation of lipophagy might point to possible therapeutic strategies for combating a broad range of liver diseases and atherosclerosis.

Autophagy is generally downregulated in high fat diet models [102]. Disturbances in hepatic autophagy in obese mice induce insulin resistance through the promotion of endoplasmic reticulum stress. Restitution of autophagy through overexpression of Atg7 in these mice can restore insulin levels back to normal and improved glucose tolerance. Excessive lipid droplets deposition in steatosis developed in alcoholic fatty liver disease, presenting as a common symptom of alcohol abuse, which can be treated more efficiently using new approaches regulating lipophagy in liver cells [103]. LAL insufficiency is the cause of Wolman disease and cholesterol ester storage disease; however, the contribution of decreased activity of lipophagy to LD accumulation is not known.

## 8. Some Perspectives of Prevention and Treatment of Dyslipidemia by Polysaccharides in Medicine

Traditional Chinese medicine has used many polysaccharide-containing herbs, which regulate triglyceride and cholesterol metabolism, and these molecules might be used for clinical treatment. Several mechanisms of hypolipidemic effects of these natural herbs were presented and analyzed in recent review [106].

In connection with recent results on lipophagy activation, new approaches are important in the treatment of steatohepatitis [105,109], cardiometabolic syndrome [110], diabetes, and other diseases.

Some protective effects of various polysaccharides have been shown. Newly synthesized α-galacto-oligosaccharide mixture (α-GOSg), 0.5% in drinking water, revealed positive effects on high-fat/western-style diet-induced metabolic abnormality in mice [111]. Mice treated with α-GOSg mixture had significantly lower body weight and body fat (*p* < 0.05). In addition, they have significantly reduced serum levels of total cholesterol and LDL-cholesterol, alanine aminotransferase activity, and liver lipids.

## 9. Conclusions

Emerging evidence suggesting that nutrition-driven changes affect cholesterol levels and have general effects on lipid metabolism led us to the present review. We presented data on several polysaccharides (β-glucan, CMG, mannans A and B, and fucoidan) that have hypolipidemic effects, with specific features in each case. Until now, some polysaccharides have been used mainly as food supplements but seem to hold promise for broad applications in the clinic. At the same time, it is important to note that due to their pleiotropic effects, it remains difficult to clearly establish the mechanisms of action. Further research is required to better understand the mechanism. Based on our review, it is clear that each polysaccharide uses different mechanisms of action, but at the same time, promotes more physiologically-based rebalancing of the level of lipids, than statins blocking the action of cholesterol-producing enzymes in the liver.

## Figures and Tables

**Table 1:** Serum total-cholesterol and TG levels (mg/dl) in mice that received either Triton WR 1339 (500 mg/kg, i.p., single dose) or poloxamer 407 in the same dose and mode of administration (M ± m, N = 10).

Parameter	Control	Triton WR 1339	Poloxamer 40
Cholesterol	108.4 ± 11.6	189 ± 11.6 *	232.2 ± 2.3 *^,#^
TG	115.1 ± 8.8	1610.7 ± 155.8 *	2283.3 ± 88.5 *^,#^

* *p* < 0.001 vs. control; ^#^ *p* < 0.001 vs. the Triton WR 1339 group.
